# Lead in the Treatment of Phthisis

**Published:** 1861

**Authors:** 


### LEAD IN THE TREATMENT OF PHTHISIS.

Remedies for phthisis, if recommended with the least show of authority, are always eagerly sought for. The number of cases of recovery from tubercular disorganization of the lungs is sufficient to keep alive in the minds of the professional, as well as unprofessional public, the hope that some cure may at last be found for it. By the London *Lancet* for August 17th, we see that M. Beau places some confidence, at the present time, in the use of lead in the treatment of this disease. Its Paris correspondent writes, in speaking of the last clinical lecture of M. Beau—

"Phthisis," he said," "has been cured by all sorts of methods and in all sorts of ways; the fact being that, as this malady depends upon the reciprocal action of a diathesis and of globular anæmia, the remedy which is able to oppose the progress of the latter condition may not only prevent the disease from making further inroads on the system, but also contribute to the healing of such lesions as already exist. My intention is not certainly to enumerate all the means for treating consumption which have in turn been extolled and employed against this malady; you will find them detailed at length in the treatise of Baumes, of Montpellier, published in 1805. I shall limit myself to a few." After mentioning sea voyages, horse exercise, southern climates, emetics, chloride of sodium, asses' milk, Iceland moss, conserve of roses, quinine, fir-sprouts, watercress, sulphur, sulphureous waters, cod-liver oil, iodine, turtle-soup, snail-broth, oysters, strawberries in great quantities, hydrotherapy, the hypophosphites, and steel, the lecturer at last arrived at his favorite remedy—lead. "The employment of lead," he said, "in the treatment of tubercular consumption is not new. The idea is a borrowed one. What is new, is the manner in which I administer it, and in which I have understood its action. I must first tell you how I was in the onset induced to employ it. Starting from the theory, which I hold in common with many other sbservers, that the development of tubercle is favored by the anæmic state, I had been struck by the fact that the workmen employed in establishments where lead is much handled, although anæmic and cachetic to the last degree, very rarely present symptoms of tubercular deposit in the lungs.

During a period of six or seven years at the Hospital Cochin, amongst the many patients whom I treated for lead poisoning, I never saw a single case of phthisis. Once only, in the lungs of a house-painter, I met with tubercle; but I subsequently ascertained that this man had never had any of the signs of the saturine affection. I went a step further, and inquired into the diseases usually met with in the trades habitually working with lead, and found that cough among operatives of this class was of rare occurrence. I began then to think within myself that if lead could prevent, it might, also very possibly cure, phthisis, and was much tempted to make the experiment, but waited for further evidence, and was presently emboldened by an accidental occurrence. Shortly after my entry into the service of the Charite, a man, affected with lead colic, fell under my observation. On examining his chest I found evident symptoms of tubercular deposit; and on inquiry, ascertuined that the patient had been consumptive for years. Finding himself without work, this man had taken a job at the lead works at Clichy, and there had caught his colic. He assured me that since his seizure both cough and expectoration had diminished; in fact, so much so as to have made him think it useless to direct my attention to the state of his lungs. He left the hospital some time afterwards, cured of his colic, and with considerable improvement of the pulmonary symptoms. In the case of a second patient, who shortly afterwards entered my wards, I was informed that the signs of consumption, held in abeyance during repeated attacks of lead colic, had reappeared each time that the latter malady had been removed. I endeavored, on the verification of this fact in my hospital service, to reproduce the saturnine when the phthisis seemed inclined to gain ground, and succeeded beyond my expectations in arresting its progress on several occasions. Such were the incidents which led me to the adoption of my present method of treatment, and *the results I have obtained are not of a nature to justify me in laying the method aside.* The following is the manner in which I employ this drug: I begin by administering an emetic, in order to arouse the stomach from the state of torpor which it is so common to meet with, either as cause or effect, in tubercular phithisis. After the lapse of a week or thereabouts, I commence the lead (I prefer the carbonate, as being better borne by the stomach than the acetate), in pills of two grains each. The dose is taken at first once a day, then twice, and so on, until six pills, or twelve grains of the lead, are swallowed within the course of twenty-four hours; the best time for administration being before each repast, as the drug is that moment less likely to cause vomiting. This treatment must be continued until a sufficient saturnine impregnation has taken place, a generous diet being allowed the patient at the same time."

M. Beau has added an appendix to his two former lectures during the course of the week, which, as it completes his previous observations on the *lead* treatment of phthisis, I think I may interest your readers by recording.

"With regard to the first effects," he says, "noticeable on the employment of lead in dealing with consumption, the most favorable is the amelioration of the cough and expectoration. Sometimes at the end of two, at others at the eud of eight or ten days (the difference of time depending on individual peculiarity), the cough, from being incessant, becomes less frequent, and allows the sufferer some respite, enabling him to sleep. The expectoration, from being abundant and purulent in nature, diminishes and becomes less copious, and of a mucous character. This is not all; you will remember one particular character of the tubercular sputum, long ago recognized by Fouquier—namely, that in pasing from the larynx to the mouth, it almost invariably, by virtue of a specially irritating property it possesses, irritates the pharynx, and produces an effort of vomiting. Another mark of the progress and success of the saturnine treatment, is precisely the cessation of this retching during expectoration. At a variable period, also, and consequent upon the amelioration in the cough and sputa, the physical signs furnished by auscultation likewise bear testimony to diminished activity in the pulmonary suppuration. The subclavicular pains diminish, and can hardly be recalled by pressure with the finger, and the nocturnal hectic fever very generally disappears entirely. I say the *nocturnal* fever advisedly; for you know the grave prognostic which attaches to the continued form of fever when occurring in this disease. With regard to the *physiological* effects of lead on the system, the first remarked is, for the most part, loss of appetite. There are, however, certain cases in which the administration of this drug seems rather to excite than to repress the digestive functions and desire for food, and such are certainly the most favorable; for here, in addition to the advantages afforded by the special action of the medicine, we have the concurrence of an active nutrition. Moreover, it is to be noted, that those patients in whom the functions of the stomach remain undisturbed, enjoy perfect immunity from the poisonous effects of the remedy. Such cases are, however, too rare to be counted on. In addition to the loss of appetite, gastralgia very generally supervenes, and occasionally, but rarely, vomiting, dry cough, and dyspnœa, the latter symptoms being exclusively of a gastric origin, and unconnected with increase of morbid action in the lung.—The colic produced by the administration of lead for a therapeutical purpose differs from that witnessed in workmen who have incurred saturnine intoxication in the exercise of their trade, in one important particular—namely, that whilst in the first diarrhœa is very generally induced, in the second case obstinate constipation for the most part exists. The blue line on the gums is another effect of the lead treatment, and appears from the tenth to the fifteenth day. Some patients there are, however, who, though presenting the other indication of saturnine saturation, are without this particular sign of constitutional affection. I believe that the blue line is only seen when there is some superficial erosion or ulceration of the margins of the gum. The predominating symptom of the therapeutical intoxication and that which is the most constant of all, is pain occurring in certain portions of the body. This pain, which appears at variable periods during the treatment, as early sometimes as the second, and rarely later than the tenth day, is occasionally present in the trunk, but mostly in the arms and legs. It is either of a shooting, darting description, or may resemble the pressure of a heavy weight, or the constriction of a metalic ring round the limbs. The physician at the same time must be careful to distinguish between the pains symptomatic of phthisis and those induced by the treatment." M. Beau likewise informs us that he never witnessed either epilepsy or those other brain symptoms occasionally met with amongst lead-workers, and ends by saying that he believes the cases of phthisis are very rare in which a saturnine treatment will not be found useful in either arresting or retarding the progress of the disease.

